# Postnatal treatment and evolution patterns of giant fetal hepatic hemangioma: a case series of 29 patients

**DOI:** 10.1186/s12887-023-04476-2

**Published:** 2024-01-03

**Authors:** Lu-lu Xie, Yan-bing Huang, Kui-ran Dong, Shao-bo Yang, Chun Shen, Yang-yang Ma

**Affiliations:** 1 Shanghai Institute of Infectious Disease and Biosecurity, Shanghai, 200032 China; 2 College of Clinical Medicine for Obstetrics & Gynecology and Pediatrics, Fujian Children’s Hospital, Fujian Medical University, Fuzhou, 350001 China; 3 Department of Pediatric Surgery, Shanghai Key Laboratory of Birth Defect, Key Laboratory of Neonatal Disease, Ministry of Health, Children’s Hospital of Fudan University, 399 Wan Yuan Road, Shanghai, 201102 China; 4 Department of Pathology, Children’s Hospital of Fudan University, Shanghai, China

**Keywords:** Congenital hepatic hemangioma, Prenatal diagnosis, Prenatal diagnosis, Ultrasound

## Abstract

**Objectives:**

To explore the clinical characteristics, postnatal treatment and prognosis of giant fetal hepatic hemangioma (GFHH).

**Method:**

Retrospective analysis was performed on children with giant fetal hepatic hemangioma (maximum tumor diameter > 40 mm) diagnosed by prenatal ultrasound and MRI from December 2016 to December 2020. These patients were observed and treated at the Children’s Hospital of Fudan University after birth. The clinical data were collected to analyze the clinical characteristics, treatment, and prognosis of GFHH using independent sample t tests or Fisher’s exact tests.

**Results:**

Twenty-nine patients who were detected by routine ultrasound in the second and third trimester of pregnancy with giant fetal hepatic hemangiomas were included. The first prenatal ultrasound diagnosis of gestational age was 34.0 ± 4.3 weeks, ranging from 22 to 39 weeks. Of the patients, 28 had focal GFHHs and 1 had multifocal GFHHs. Surgery was performed, and the diagnosis was confirmed histopathologically in two patients. There were 8 cases with echocardiography-based evidence of pulmonary hypertension, 11 cases had a cardiothoracic ratio > 0.6, and 4 cases had hepatic arteriovenous fistula (AVF). The median follow-up time was 37 months (range: 14–70 months). During the follow-up, 12 patients received medical treatment with propranolol as the first-line therapy. The treatment group had a higher ratio of cardiothoracic ratio > 0.6 (*P* = 0.022) and lower albumin levels (*P* = 0.018). Four (14.8%) lesions showed postnatal growth before involuting. Complete response was observed in 13 (13/29) patients, and partial response was observed in 16 (16/29) patients.

**Conclusions:**

Fetal giant hepatic hemangioma is mainly localized, and its clinical outcome conforms to RICH (rapidly involuting) and PICH (partially involuting), but some fetal giant hepatic hemangiomas will continue to grow after birth and then gradually decrease. For uncomplicated giant fetal hepatic hemangioma, postnatal follow-up is the main concern, while those with complications require aggressive medical treatment. Propranolol may have no effect on the volume change of GFHH.

## Introduction

Fetal hepatic hemangiomas (FHH) are the most common benign vascular neoplasms, accounting for approximately 60% of fetal liver tumors [1]. Previous studies have suggested that FHH proliferates in utero and generally reaches peak size prior to or at birth. Three subtypes of congenital hemangiomas have been further described based on their evolution pattern: rapidly involuting (RICH), partially involuting (PICH), and noninvoluting (NICH) congenital hemangiomas [2, 3]. Giant fetal hepatic hemangiomas (GFHHs), defined as tumors measuring more than 40 mm in diameter [4], are rare and can be associated with several life-threatening complications, such as high-output cardiac failure, consumptive coagulopathy, thrombocytopenia, and intratumoral bleeding. The prevalence of GFHH has been calculated as 0.64/10,000 pregnancies [5], and the clinical presentation is highly variable, from asymptomatic to life-threatening [6]. The literature about GFHH consists mainly of relatively small case series, and thus, there is a lack of understanding of the treatment and clinical outcomes. The aim of this study was to investigate the clinical characteristics, optimal postnatal treatment strategy and regression of fetal giant hepatic hematoma by reviewing the clinical data of the children.

## Materials and methods

### Data collected

We retrospectively reviewed all cases of GFHH (maximum tumor diameter > 40 mm) diagnosed at or referred to our tertiary referral center by antenatal ultrasound between 2016 and 2020 with a follow-up period of > 1 year.

Records were reviewed prenatally and postnatally.

1. Prenatal data: age at diagnosis, mode of delivery and size of the lesion.
2. Postnatal data: sex, gestational week, birth weight, cutaneous hemangiomas, complications, imaging (prenatal ultrasound and MR, postnatal serial ultrasound, CT and MR, cardiac ultrasound, chest X-ray), laboratory data (routine blood, coagulation function, liver function, AFP, thyroid function), and pathology data.

Age at diagnosis was assessed as the age on the date that hepatic lesions were first confirmed by imaging. Tumor volume changes over time were quantified by volumetric analysis of GFHH, which was based on US and cross-sectional imaging (CT and MRI). Lesion volumes were estimated using the standard ellipsoid formula, width× height× length × 0.52.

### Management

**Fig. 1:**
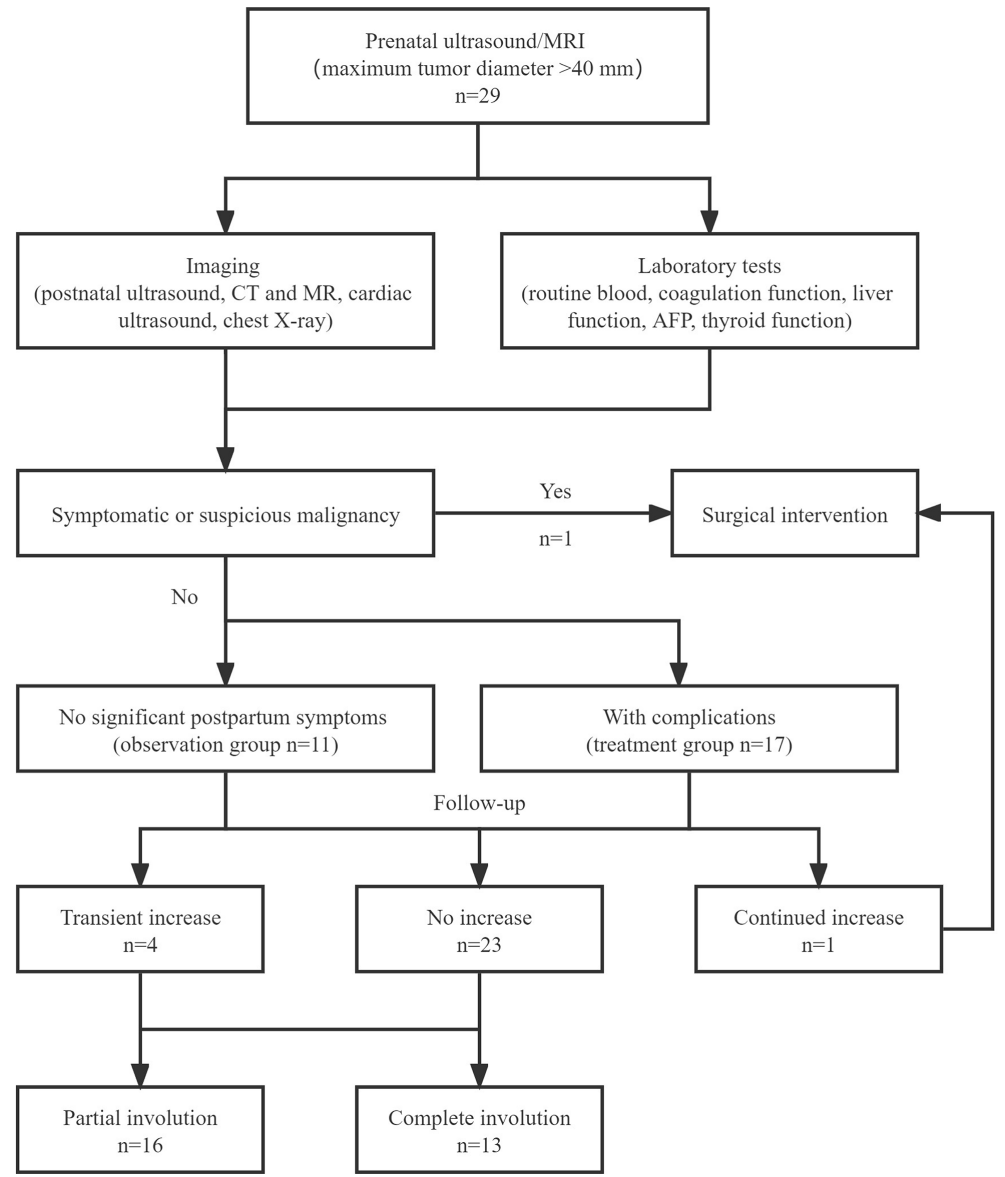
Algorithm for GFHH management

The management of GFHH is based on clinical presentation and includes observational, medical, surgical, and radiological interventional treatment options (Fig. 1).

Diagnosis: The finding of a unifocal congenital liver mass and suspicion of CHH by clinical history and imaging would initially determine conservative treatment and follow-up with serial ultrasounds in asymptomatic patients. When an expert panel of radiologists experienced in liver disease imaging was not able to conclude on a clear diagnosis or when growth of the lesion was determined in consecutive controls, a biopsy was performed to rule out malignancy, which led to definite diagnosis. CHHs were then classified into three groups according to the lesion range on postpartum.

Imaging: focal type, multiple types and diffuse type.

Treatment: Children with no significant postpartum symptoms were observed conservatively (observation group); children with complications such as postnatal shortness of breath, vomiting due to abdominal distension, and cardiac insufficiency were treated (treatment group). Children in the treatment group were treated with propranolol as the first-line treatment, starting at 0.5 mg/(kg-d) and increasing to 2 mg/(kg-d) for at least six months. In combination with cardiac insufficiency, cardiac diuretics were used to improve pulmonary hypertension. In the case of combined anemia, platelet depletion, and significant coagulation abnormalities, methylprednisolone is used in combination for a short period of time, and the dosage is gradually reduced. If there is no significant response to drug treatment and postnatal observation reveals that the hepatic hemangioma is significantly enlarged, interventional treatment is recommended.

Follow-up: The follow-up information was obtained by consulting the outpatient follow-up medical records and telephone follow-up, including imaging examinations such as B-ultrasound or CT, physical examination, growth and development of the child, and survival conditions. The last follow-up was in December 2022.

### Data analysis

The data were analyzed by IBM SPSS 22.0. Measurement data conforming to a normal distribution are expressed as (®± s), and paired-samples t tests were performed for intragroup comparisons and independent t tests for intergroup comparisons. Data that did not conform to a normal distribution were represented by the median (min ~ max), and a nonparametric test was used for comparisons between groups. Count data are expressed as the number of cases and percentage (%), and the χ2 test or Fisher’s exact probability method was used for comparisons between groups. P < 0.05 was considered statistically significant. The graphs, calculations and statistical analyses were performed using GraphPad Prism software version 9.0 for Mac (GraphPad Software, San Diego, CA).

## Results

### Patient characteristics

#### Prenatal diagnosis

**Fig. 2:**
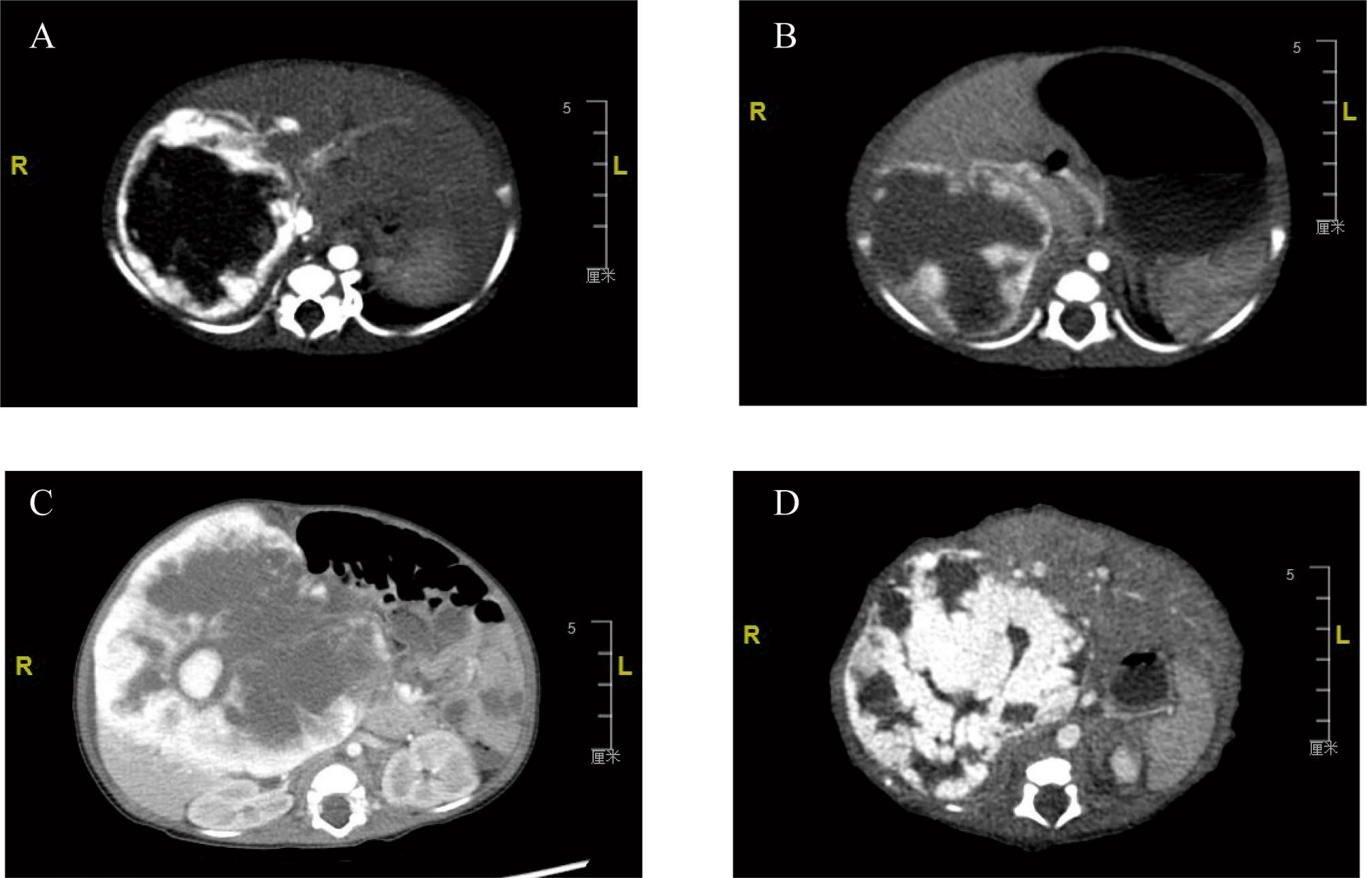
Computed tomography (CT) images of the observation group (**A, B**) and treatment group (**C, D**)

All 29 children were identified by routine prenatal ultrasound, and three were further identified by prenatal MRI. After birth, they all received ultrasound and CT (or MR), and the diagnosis was finally confirmed by typical imaging (Fig. 2) in 27 cases and by histopathology in two cases. The first prenatal ultrasound diagnosis of gestational age was 34.0 ± 4.3 weeks, ranging from 22 to 39 weeks.

### Perinatal

Among the 29 patients, 18 (62%) were male, and 11 (38%) were female. All were delivered at term, with a gestational age of 38.6 ± 1.4 weeks (range: 36 weeks + 6 ~ 41 weeks) and birth weight of 3181 ± 310 g (range: 2650 g ~ 4000 g). Nineteen cases (66%) were delivered vaginally, and 10 cases (34%) were delivered by cesarean section, among which three cases were delivered by cesarean section due to a significant increase in the fetal cardiothoracic ratio, fetal heart failure and excessive amniotic fluid caused by tumor factors. One case was delivered due to a rapid increase in tumor size, and the remaining six cases were delivered due to other obstetric factors.

### Neonatal period

Of the 29 children, 25 were referred to our hospital immediately after birth, and the remaining four had a history of previous visits to other hospitals. The age at postnatal presentation was 17 ± 13 days. Five children had varying degrees of heart failure immediately after birth; two had cutaneous hemangiomas on the left or right eyelid.

#### 1) Imaging features

The maximum diameter of the first postnatal ultrasound was 6.4 ± 1.8 cm. Twenty-eight neonates had solitary lesions, whereas only one had multiple lesions combined with splenic and retroperitoneal hemangioma. Twenty-two lesions were located in the right lobe of the liver, four lesions in the left, and three lesions at the junction. There were 8 cases with echocardiography evidence of pulmonary hypertension, 11 cases had a cardiothoracic ratio > 0.6, and 4 cases had hepatic arteriovenous fistula (AVF).

#### 2) Laboratory testing

Four neonates had elevated TSH, four had anemia, six had thrombocytopenia, 19 had coagulation abnormalities and 19 had elevated liver enzymes. Albumin levels were 34.0 ± 4.1 g/l, and methemoglobin levels were 48,209 ± 36,537 ng/ml. Demographics and clinical features of the patients are shown in Table 1.

**Table 1:** Demographic and Clinical Characteristics of the Study Population

Study cohort			Conservative	Treatment	
Characteristics		(n = 29)	(n = 15)	(n = 12)	*P* value
Sex(female)		11/29(38%)	7/15(47%)	3/12(33%)	0.42
Prenatal diagnosis gestation (wk)		34 ± 4.3	33.6 ± 4	34.8 ± 4.8	0.56
Birth gestation (wk)		38.6 ± 1.4	37 ~ 41	37 ~ 40	0.28
Birth weight (g)		3,181 ± 310	3,200 ± 355	3,185 ± 262	1.00
visiting ages(d)		17 ± 13	1–30	1–30	0.87
**Delivery pattern**					0.71
Normal labor		19(66%)	10(67%)	7(58%)	
Cesarean section		10(34%)	5(33%)	5(42%)	
**Postnatal Initial ultrasound**					0.10
Tumor size (cm)		6.5 ± 1.7	6.0 ± 1.1	7.2 ± 2.1	
**Location**					0.81
Right lobe		22(76%)	12(80%)	8(67%)	
Left lobe		4(14%)	2(13%)	2(17%)	
Right and left lobes		3(10%)	1(7%)	2(17%)	
**Cardiothoracic ratio(>0.6)**		11/21(52%)	3/8(38%)	8/11(73%)	**0.02**
**Clinical features**					
Cutaneous hemangioma		2/29(7%)	0/15	2/12(17%)	0.19
Abdominal syndrome	compartment	3/29(10%)	0/15	3/12(25%)	0.08
Hepatic arteriovenous fistula (AVF)		4/29(14%)	0/15	4/12(33%)	0.03
Congestive heart failure		5/29(17%)	0/15	5/12(42%)	0.01
Pulmonary hypertension		8/29(28%)	1/15(7%)	7/12(58%)	0.01
Thrombocytopenia		6/29(21%)	1/15(7%)	4/12(33%)	0.14
Elevated TSH		4/18(22%)	1/7(14%)	3/11(27%)	1.00
Anemia		4/29(14%)	1/15(7%)	3/12(25%)	0.29
Coagulation abnormalities		19/26(73%)	10/14(71%)	9/12(75%)	1.00
Elevated liver enzymes		19/23(83%)	11/12(92%)	7/9(78%)	0.55
Albumin(g/l)		34.0 ± 4.1(n = 23)	34 ~ 40(n = 12)	25 ~ 40(n = 9)	**0.02**
AFP(ng/ml)		48,209 ± 36,537 (n = 21)	52,036 ± 31,672 (n = 12)	52,275 ± 42,924 (n = 7)	0.99

### Group management results

#### Clinical features

All were evaluated by using postnatal color Doppler ultrasound and contrast-enhanced computed tomography (CT) or MRI. Twenty-seven patients with focal hepatic hemangioma diagnosed by typical imaging findings were divided into an observation group and a treatment group. Fifteen children received follow-up observation only (conservative group), and 12 children received drug treatment (treatment group). There was no significant difference between the 2 groups in terms of sex, age at diagnosis, mode of delivery, gestational week, birth weight, age at consultation, thrombocytopenia, anemia, TSH, liver enzymes or AFP. Cardiothoracic ratio (> 0.6) and lower albumin were significantly more frequent in patients in the treatment group. (Fisher exact test and Kruskal‒Wallis test, *P* = 0.022 and *P* = 0.018, respectively).

#### Follow-up and outcome

**Fig. 3:**
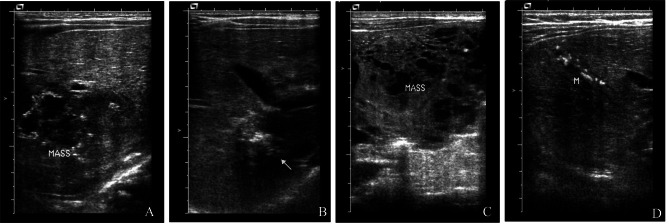
Ultrasound (US) images of the observation (**A, B**) and treatment groups (**C, D**) at birth (**A, C**) and 2 years old (**B, D**)

**Fig. 4:**
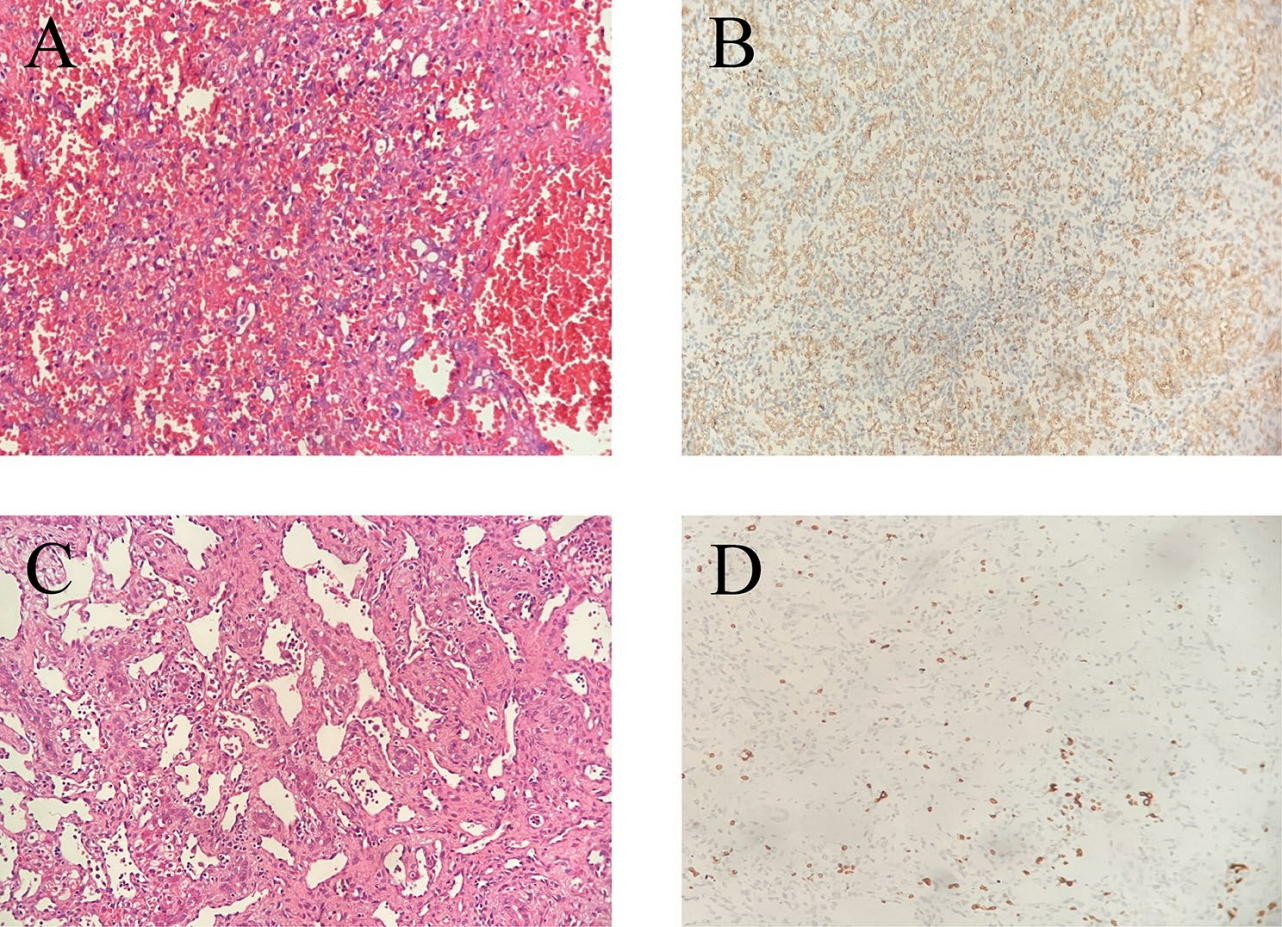
Histopathologic images of GFHHs

**Fig. 5:**
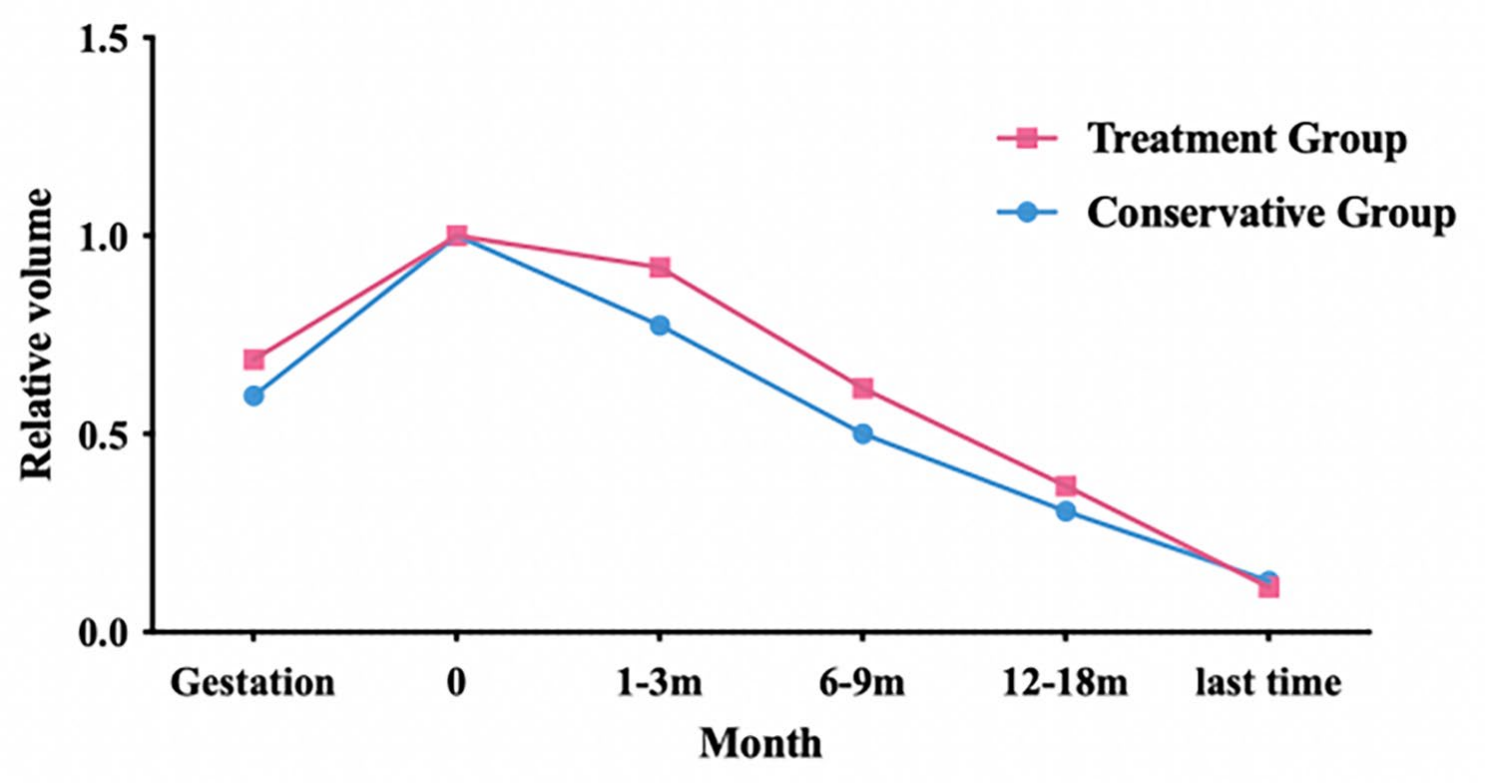
The lesions in four patients showed postnatal growth before involution (**A**). Time evolution curve of tumor volume in the treatment group and observation group (**B**)

The median follow-up period was 3.1 years (range: 1.2–5.8 years), and all children survived. All lesions in our cohort eventually decreased in size, with complete or partial regression (Fig. 3). Two of the surgically treated cases completely regressed at 3 months (Fig. 4A and B) and 7 months of age (Fig. 4C and D), while four of the remaining 27 cases showed continued growth after birth (Fig. 5A; Table 2). Eleven cases eventually showed rapid involution (leaving small calcified remnants), and 16 cases showed partial involution (Fig. 5B). The conservative group was followed up for a median of 3.4 years (range: 1.3 ~ 5.8 years), and none of them received medication for significant lesion enlargement or clinical symptoms. Seven children in this group had rapid involution, and the remaining eight had partial involution. The treatment group was followed up for a median of 2.9 years (range: 1.2 ~ 5.8 years). All 12 children received oral propranolol, and 6 of them were treated with a combination of cardiac diuretics early in the course of treatment due to combined pulmonary hypertension. One of them underwent interventional treatment (4 ml iodine oil + 6 mg bleomycin) at the age of 3 years, which did not completely regress at the age of 4 years. In addition to the interventional children, the lesions eventually involuted completely in 4 children at the age of 3–5 years and partially in the remaining 7 children. There was no statistically significant difference in prognosis between the two groups.

Histopathologic study showed variable-sized luminal structures lined with flat cells, and erythrocytes were seen in the lumen (original magnification, 40×)(A and C). The tumor cells were diffusely negative for GLUT-1 immunohistochemically. (B and D, original magnification, 200×)

**Table 2:** Clinical and imaging features of congenital hemangiomas that exhibited postnatal growth before involuting (n=4)

P	Sex	Birth gestation	Delivery pattern	Initial volume	Maximum volume	Complication	Treatment	Involution (age)	Residual lesion
P1	Male	37	Normal labor	50	78	None	Conservative	Complete (12)	NA
P2	Male	38	Cesarean section	31	48	ACS, Anemia, Pulmonary hypertension, CC, AVF	propranolol cardiac diuretics	Complete (40)	Calcification
P3	Male	38	Normal labor	50	168	Cutaneous hemangioma, CC	propranolol	Partial (12)	9 mL
P4	Male	40	Normal labor	10	22	CC	propranolol	Complete (18)	Calcification

P patient, Birth gestation birth gestation in weeks, Initial volume tumor volume in mL, Maximum volume maximum volume in mL, Involution (age) age at involution in months, ACS abdominal compartment syndrome, CC consumptive coagulopathy, AVF hepatic arteriovenous fistula

## Discussion

This series of observations of 29 patients with GFHH is the largest yet reported and is the first to have a long enough follow-up to allow comparison between the untreated and treated groups with quantification of tumor volume over time in a large cohort of patients.

### Clinical characteristics and outcome

In our study, we present 29 cases of giant fetal hepatic hemangioma diagnosed prenatally using two-dimensional ultrasound between 22 and 39 weeks gestation, all identified as isolated in apparently asymptomatic routine second- and third-trimester scans. The most striking prenatal ultrasound feature was the incidental detection of a huge solid mass in the fetal liver. In addition, fetal MRI was also used as a diagnostic technique in three cases. Fetal MRI has been reported as an additional tool for the study of hepatic infantile hemangiomas in the fetus [7]. All were evaluated by using postnatal color Doppler ultrasound and contrast-enhanced computed tomography (CT) or MRI. Twenty-seven cases were finally diagnosed by typical imaging findings, all of which were focal type. One of these lesions was resected at age 3 months because postnatal contrast-enhanced CT and MRI suggested hepatoblastoma with AFP increase. One patient experienced thrombocytopenia and anemia, while propranolol and methylprednisolone failed to shrink the tumor. Enhanced CT at the age of 7 months showed a large malignant tumor in the abdomen and pelvis, with doubtful multiple metastases in the liver and peritoneum. Splenic cavernous hemangioma and liver hemangioma were found on postoperative pathology after splenic hemangioma resection and liver mass biopsy. Immunohistochemical staining of GLUT-1 was negative in both cases.

In general, hepatoblastomas present as a single, solid, and well-circumscribed echogenic lesion, sometimes with dysplasia and coarse dense calcification. Hepatic hemangiomas are well-defined mixed solid lesions with excessive vascularity and fine-grained calcification [8, 9]. Sometimes there is an inhomogeneous structure due to tiny echogenic foci having posterior acoustic shadowing and representing calcifications (seen in up to 36% of cases), or the range of the Doppler frequency shift peak may overlap with those malignant liver tumors previously reported in the literature due to hemorrhage, necrosis, or fibrosis [10]. When imaging is atypical and malignancy is highly suspected, biopsy is considered to confirm the diagnosis [1].

There are no previous studies suggesting whether there are sex differences in fetal hepatic hemangiomas, but recent studies from China have reported a predominance of male cases [5, 11], which is consistent with the results of this study, and the mechanism needs further investigation. Most of the infants were delivered vaginally, but three underwent cesarean section because of prenatal complications, such as high-output heart failure, cardiomegaly, edema, or polyhydramnios. Hepatic hemangiomas can occur in either or both lobes of the liver. The lesions can be solitary or multifocal and vary in size [12]. Most fetal hepatic hemangiomas are focal lesions in the right lobe of the liver, while only four cases of multiple lesions have been reported in the past [11–14].

In this study, 28 patients (96.6%) had focal lesions, 22 of which were located in the right lobe. Only one case had multiple lesions, and this child underwent splenic hemangioma resection and hepatic hemangioma biopsy because of the combination of splenic hemangioma, and postoperative pathology confirmed hepatic hemangioma that was GLUT1-negative. Sepu et al. presented 45 cases detected prenatally before 2021. Almost all the reports described progressive growth in the prenatal period and gradual shrinkage in the postnatal period [1, 6]. Except for two resected lesions, all lesions in our cohort eventually decreased in size, with 11 cases of complete and 16 cases of partial regression, such that no lesion could be categorized as a noninvoluting pattern in which the clinical outcome conformed to RICH and PICH.

### Optimal treatment strategy

Observational management is advised for 15 cases, which are usually asymptomatic. Serial U/S follow-up observation is recommended to document the changes in tumor size and expected regression of the lesion. This is consistent with previous recommendations for the treatment of hepatic hemangiomas [15].

Treatment of hepatic hemangioma depends not only on the size of the tumor but also on clinical symptoms and laboratory findings. Children with clinical symptoms (such as dyspnea and abdominal distention leading to recurrent vomiting), postnatal cardiac ultrasound suggesting cardiac insufficiency, hepatic arteriovenous leakage, and significant abnormalities in coagulation received pharmacological treatment after definitive diagnosis. Previous studies have shown that GFHH may present with serious complications such as high-volume congestive heart failure, severe anemia, or KM syndrome. Without clinical intervention, the perinatal morbidity and mortality rate can be as high as 90% [16].

Clinical behavior can be innocuous or life-threatening. Thrombocytopenia, coagulation disorders, and increased liver enzymes at diagnosis seem to be the main predictors of mortality [17]. Congenital arteriovenous intrahepatic fistulas, which are hepatic hemangiomas and arteriovenous malformations (AVMs), are rare [18]. Both vascular anomalies were confused with each other. However, vascular anomalies of neoplastic origin show an increase in the cell proliferation of the soft tissue mass, whereas AVMs do not. Similarly, both hepatic hemangiomas and hepatic AVMs have nourishing dilated arteries and draining dilated veins [19]. As a result, high output heart failure, hepatomegaly, consumption coagulopathy, and hemorrhage are common complications in both neonatal periods [20]. Prophylactic treatment with steroids and propranolol was given to four children with giant hepatic hemangiomas complicated by arteriovenous fistulas; eventually, two patients showed complete regression, and two patients showed partial regression.

In this study, six patients with hypertension used cardiac diuretics in the early stage of treatment. For children with high volume congestive heart failure, severe anemia, thrombocytopenia, or KM syndrome in the early stage, methylprednisolone was used in combination and was gradually discontinued when the platelets were monitored and normalized and the condition stabilized.

Our previous study showed that corticosteroid therapy combined with active treatment for congestive heart failure and pneumonia was effective in most cases of neonatal hepatic hemangioma [21]. Rapid involution of approximately 30% of lesions has been reported after high-dose corticosteroid treatment, while the other 40% had a less pronounced effect, but congestive heart failure and coagulation dysfunction were relieved [22].

Although there is a lack of consensus regarding whether transcatheter arterial embolization (TAE) is effective in the treatment of hemangiomas and regarding the severity of complications, [23–25] a consensus exists among experts that interventional therapy is a viable option when drug therapy is not working [26]. Torkian et al. conducted a systematic review and meta-analysis suggesting that TAE with bleomycin, pingyangmycin, or ethanol, in combination with lipiodol, was safe and effective [23]. Yuan Bing et al. retrospectively reviewed 241 patients with hepatic hemangioma who had undergone TAE with lipiodol-bleomycin emulsion (LBE) and found that TAE with LBE was feasible and effective for giant hepatic hemangiomas in all patients. TAE was performed successfully in the participant without serious complications [27].

The postnatal growth of focal, congenital, GLUT1-negative hemangiomas has not been previously described in the liver. However, a recent study documenting the spontaneous evolution of untreated focal congenital hepatic lesions consistent with hepatic hemangioma has shown that some focal congenital lesions can show significant postnatal growth without evidence of intralesional hemorrhage [2]. “Although they disagree about the diagnostic criteria for congenital hepatic hemangioma (prenatal diagnosis and/or age at diagnosis ≤ 7 days and/or GLUT1-negative tumors), the age ranges for diagnosis of congenital hepatic hemangioma and infantile hepatic hemangioma overlap.” However, all the children included in our study were prenatally diagnosed, and four of them also had postnatal growth, one case in the observation group and three cases in the drug treatment group. Additionally, a case series in China also reported two children who were treated with propranolol after birth, and dynamic observation showed that the lesions did not subside but appeared to grow [11].

The therapeutic effect of oral propranolol administration in IHs was incidentally discovered in 2008 [28]. Propranolol is a nonselective beta-blocker that acts on both β-1 and β-2 adrenergic receptors. Oral propranolol actually represents the first-line approach for the pharmacological treatment of IHs, which suppress lesion proliferation [29–32]. Multiple cutaneous and infantile hepatic hemangiomas respond significantly to propranolol monotherapy in the neonatal period [33]. Positive staining for glucose transporter isoform 1 (GLUT 1) is the histologic marker that differentiates IHH from other types of congenital hepatic hemangioma. IHH does not spontaneously subside and responds to drugs. RICH usually subsides at the age of 12–14 months. Clinical management can be achieved through symptomatic treatment, observation, waiting, and close monitoring of hemangioma size. Drug treatment (corticosteroids, propranolol) has not yet shown efficacy [34, 35]. Considering the clinical outcome of GFHH, we believe that propranolol may have no effect on the volume change.

For uncomplicated fetal giant hepatic hemangioma, perinatal follow-up observation is the mainstay, and fetal hepatic hemangioma with comorbidities requires active medical treatment. The main limitations of this study include its retrospective and single-center nature, as well as the intra- and interobserver variability in imaging test measurements. The small sample size and selection bias are further limitations. In addition, the absence of similar studies makes it difficult to compare the results obtained. Despite its small sample size, it represents the largest reported series of patients with GFHH. However, multicenter studies are required to confirm these results.

## Data Availability

The datasets generated and analyzed during the current study are not publicly available due to limitations of ethical approval involving the subject data and anonymity, but are available from the corresponding author on reasonable request.

## Abbreviations

GFHH: Giant fetal hepatic hemangioma
AVF: Arteriovenous fistula
RICH: Rapidly involuting congenital hemangioma
PICH: Partially involuting congenital hemangioma
NICH: Noninvoluting congenital hemangioma
FHH: Fetal hepatic hemangiomas
TSH: Thyroid stimulating hormone
AFP: α-fetoprotein
GLUT1: Glucose transporter-1
AVM: Arteriovenous malformations
TAE: Transcatheter arterial embolization
LBE: Lipiodol-bleomycin emulsion
